# Nutritional resilience in Nepal following the earthquake of 2015

**DOI:** 10.1371/journal.pone.0205438

**Published:** 2018-11-07

**Authors:** Andrew L. Thorne-Lyman, Angela K. C., Swetha Manohar, Binod Shrestha, Bareng A. S. Nonyane, Sumanta Neupane, Shiva Bhandari, Rolf D. Klemm, Patrick Webb, Keith P. West

**Affiliations:** 1 Center for Human Nutrition, Department of International Health, Johns Hopkins Bloomberg School of Public Health, Baltimore, MD, United States of America; 2 Department of Nutrition, Harvard T.H. Chan School of Public Health, Boston, MA, United States of America; 3 PoSHAN Study Team, Johns Hopkins University, Kathmandu, Nepal; 4 Department of Health Promotion, Education & Behavior, University of South Carolina, Columbia, SC, United States of America; 5 Helen Keller International, New York, NY, United States of America; 6 Friedman School of Nutrition Science and Policy, Tufts University, Boston, MA, United States of America; The Hospital for Sick Children, CANADA

## Abstract

**Background:**

The 2015 earthquake in Nepal caused massive damages and triggered relief activities to minimize human suffering. The post-earthquake nutrition and food security situation in the hardest hit areas remains uncertain.

**Methods:**

Two national cross-sectional surveys were conducted in 2014 and 2016 among households (HH) with pre-school aged children or newly married women. Of the 21 village development committees (VDCs) included in this sample, 7 fell within “earthquake-affected” areas. This paper presents data from 982 HH, 1015 women, and 883 children from 2014 and 1056 HH, 1083 women, and 998 children from 2016 living in these areas, with longitudinal overlap of about 55%. Prevalence estimates and 95% confidence intervals were calculated, and logistic regression was used to calculate p-values, both using robust estimates of standard errors to account for clustering.

**Results:**

From 2014 to 2016, child wasting (weight-for-height z score <-2) fell from 4.5% (95% CI 3.3%– 6.1%) to 2.1% (1.4%– 3.1%) and food insecurity (assessed using the household food insecurity access scale) dropped from 17.6% (11.7%– 25.6%) to 12.4% (6.9%– 21.2%). Child stunting prevalence remained similar at both time-points. Improvements were also evident in dietary diversity and breastfeeding indicators.

**Conclusions:**

Nutrition and food security conditions remained comparable or improved one year after the earthquake despite evidence of structural and other damage. Livelihood resilience to shocks and/or effective nutrition, food or health interventions may have helped buffer the impact on nutrition, although this hypothesis requires further exploration.

## Introduction

The 2015 Nepal earthquake and its subsequent series of aftershocks caused widespread damage and loss of life. In its immediate aftermath, around 9,000 people died, 22,000 were injured, 2.6 million displaced, and over 750,000 houses were damaged [1, 2]. Over USD 7.1 billion was lost in infrastructure and the productive sectors [1]. Households in agriculturally and economically vulnerable locations in the Mountains and Hills were the hardest hit, suffering extensive losses of agricultural assets, tools, livestock shelters, irrigation infrastructure and productivity [1, 3]. More than 50% of households in the six hardest hit districts lost their stored grains and seeds, 20% lost cattle and 42% lost their poultry [3].

At the request of the Government of Nepal, humanitarian clusters were organised to coordinate emergency responses across different sectors. Upon evaluation of immediate damages, the government identified 31 districts as “earthquake-affected”, among which 14 were “crisis-hit” and were prioritised to receive aid and rehabilitation assistance [4, 5]. The Nutrition Cluster, composed of 28 actors under the Ministry of Health and Population (MoHP), identified several interventions in need of scale-up to prevent a deterioration of the situation, including breastfeeding, infant and young child feeding (IYCF) promotion, and therapeutic feeding [6]. The Food Security Cluster distributed 17,000 metric tons of food during the first five months after the earthquake [7].

Reconstruction efforts following the earthquake were reportedly slow and inconsistent, partly because financial resources were scarce, and disbursement was slow [4, 8]. However, to our knowledge, there has been no quantitative assessment of the post-earthquake household-level food security and nutritional situation published to date in the peer review literature. Moreover, a recent review of 152 studies identified a more general gap in the knowledge about the longer-term impacts of earthquakes in low and middle-income countries [9]. The present study makes use of same-survey national data collected in Nepal from 2013 to 2016 among households with young children or newlywed couples [10–13]. Specific objectives of this analysis were to (1) assess the change in prevalence of child stunting and wasting in a *post hoc* sample of areas classified by the government as “earthquake-affected” from 2014 to 2016 (2) examine in greater detail the shocks experienced by households, potential risk factors for malnutrition, and indicators of household resilience in the same sample.

## Materials and methods

### Study design

This analysis uses data from two rounds of the PoSHAN Community Study, a nationally representative, mixed-longitudinal sequence of annual surveys from 2013 through 2016, designed to assess household agricultural practices, socioeconomic status, food security, and diet, health, and nutritional status of preschool children and women of reproductive age [10]. Data from mid-2014 provide an overview of the condition in the year preceding the April-May 2015 earthquake in Nepal, and the condition approximately one year after the disaster in mid-2016 [13]. As the quake struck the country shortly before the scheduled mid-2015 survey was to begin, the survey was not administered in earthquake-affected areas due to the need to redirect available resources to humanitarian services and difficulties in mounting survey teams to reach the Hills or Mountains.

The design of the overall study is described in detail in previous papers [10–13]. Following a contiguous listing of village development committees (VDC’s) from west to east within each of the three agro-ecological zones of Nepal (mountains, hills and Terai), a systematic sampling approach was used to select 21 VDC’s, seven from each zone. Within each VDC, three of nine administrative wards were randomly drawn and all households were visited and enumerated. Households with children <5 years or recently married (within the past two years) nulliparous women were consented and invited to participate in a full assessment protocol. Each year, field teams returned to the same areas, and followed the same household enumeration, consent, and assessment procedures. In surveys from 2014–16, previously enrolled children <5 years were eligible to be followed until 72 months of age, after which they were censored from study. Identifiers from households visited in both surveys were linked to form a longitudinal dataset to augment cross-sectional analyses. This cohort dataset excluded households lost to follow-up in 2016 due to movement, refusal or death, and those in which the indexed preschool child had aged out of eligibility and no new children had been born.

### Definition of earthquake-affectedness

**Fig 1:**
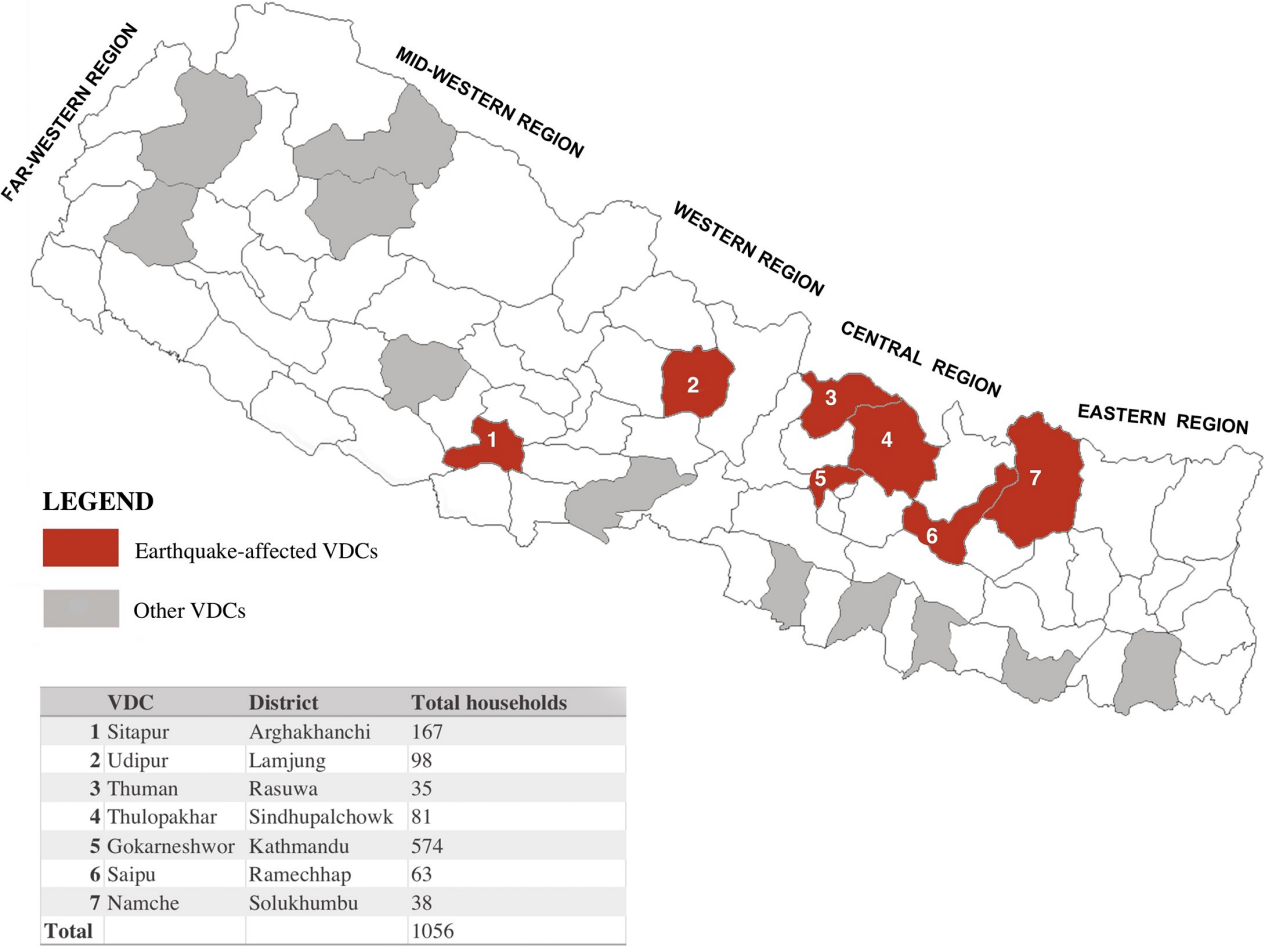
Map of PoSHAN Community Study nationally sampled survey sites (VDCs) that were affected by the 2015 earthquake.

Following the earthquake, the Government of Nepal categorised 31 out of 75 districts as “earthquake-affected,” and prioritised a subset of 14 districts, termed “severely-hit” or “crisis-hit”, for relief assistance. Our national sample included seven VDC’s across the Hills (n = 4) and Mountains (n = 3) from districts that had been categorised as earthquake-affected, including in Solukhumbu, Rasuwa, Sindhupalchok, Arghakhanchi, Lamjung, Kathmandu and Ramechhap (**Fig 1**) [5]. The first four of these were prioritised for assistance, the next two were categorized as ‘hit with heavy losses” and the final VDC was “slightly affected” respectively. In this paper, we examine nutrition and food security trends within this *post hoc* “earthquake-affected” sample. We focused on the subset of affected areas because prior studies had found greater adverse effects on nutrition closer to earthquake epicenters and because of the desire to better understand the outcomes that may have been influenced by relief and rehabilitation activities [14–15].

### Survey content and measurements

Data collected at both time-points included questions about socioeconomic and demographic characteristics of households, agricultural production in the prior year, land and animal ownership, a dichotomous food security indicator created using the Household Food Insecurity Access Scale (HFIAS) [16], and the Minimum Dietary Diversity for Women (MDD-W) indicator, a ten-item women’s dietary diversity score reflecting consumption in the prior day and dichotomized at ≤5 food groups [17]. Questions about infant and young child feeding practices were asked at both rounds in accordance with the WHO IYCF indicator guide, with an additional question about colostrum given during early infancy [18]. A composite wealth index was calculated from the national sample with variables related to household asset ownership and other socio-economic factors using principal component analysis, and categorised into quintiles for this analysis [19].

In both rounds, households were also asked to recall any shocks experienced the previous year, including death of a family member, having a family member suffer injury or morbidity, job loss, business loss, structural/house damage, crop loss, and livestock or poultry loss. A post-earthquake module administered only in 2016 also asked the households that had reported a shock whether the shock was due to the earthquake and the degree to which they had recovered from each shock (response options were: fully, partly or not at all).

Anthropometric measures were taken in triplicate by trained staff. Weight was measured to the nearest 100 g using a digital scale (Seca Scales, Columbia, MD). Supine length was measured to the nearest 0.1 cm for infants 0–23 months and standing height for children 24–59 months, accepting the median of triplicate readings as the true value. Weight and length/height measures were converted to z-scores of weight-for-height using the WHO growth standards, with a cut-off of <-2 defining moderate to severe, and <-3 as severe wasting. Height-for-age z-scores were computed, with a cut-off <-2 used to define stunting [20].

In each national round, blood was obtained in a random ~25% subsample of infants <6 months of age by heel stick, and children ≥ 6 months and women by finger stick for haemoglobin (Hb) analysis using a portable haemoglobinometer (Hb201, HemoCue AB, Angelholm, Sweden). Hb concentrations were adjusted for altitude as recommended by WHO [21]. Anaemia was defined using standard WHO cut-offs (Hb <110 g/L in children and pregnant women, and Hb <120 g/L in non-pregnant women), and measurements from pregnant and non-pregnant women were pooled to generate a combined prevalence estimate. Children found to be severely malnourished (mid upper arm circumference (MUAC) <11.5cm) or anaemic (Hb <70 g/L) were referred to local health posts for further evaluation and treatment.

### Data collection

Teams consisting of three interviewers and one supervisor were trained, standardised and assigned to complete field work in one VDC. Forms were collated and transported to Kathmandu for double-entry of data.

### Statistical analysis

Proportions of households experiencing various types of shocks and perceived degree of recovery were calculated. Prevalence of wasting, stunting, food security and crop production were estimated together with their associated standard errors and confidence intervals using robust estimates of standard errors to account for clustering. Logistic regression with robust standard errors was conducted to test for statistical significance of post-earthquake changes in household food security, dietary diversity and anthropometry scores from 2014 to 2016. For households with longitudinal data from both time points, those reporting each type of shock and perceived recovery were stratified by their pre-earthquake socio-economic status. Cochran–Armitage tests for trend were conducted to test for linear trends across strata. Stata version 15.1 (StataCorp, Texas) was used to conduct all statistical analyses.

### Ethical approval

Signed or verbal informed consent, due to illiteracy in the population, was obtained from head of households, newly married women, and mothers or caregivers of children <5 prior to all interviews. Ethical approval for the PoSHAN Community Studies was obtained annually from the Nepal Health Research Council (Reg. No.: 91/2016), an autonomous body under the MoHP, and the Institutional Review Board at the Johns Hopkins Bloomberg School of Public Health, Baltimore, MD (IRB number: 4937).

### Role of the funding source

The funders of this study had no role in the study design, or the collection, analysis, and interpretation of data, in writing of the report, or the decision to submit the paper for publication. The corresponding author had full access to all the data in the study and had final responsibility for the decision to submit for publication.

## Results

The study flow diagram is presented in **S1 Fig**. In 2016, 12,143 households were screened, and 5,109 of the 5,173 eligible households consented. These households included 5,568 children <5 years with approximately 14%, 25%, and 61% assessed in the Mountains, Hills and Terai, respectively. The 2014 cross-sectional sample included 982 households with 1015 women and 883 children under five years (Fig 1S). The post-earthquake (2016) sample included 1056 households, 1083 women, and 998 children, of which 537 households, 540 women, and 352 children also had data collected during 2014 and contributed to the longitudinal sample. A sub-sample of 224 women and 154 children, and 247 women and 189 children were assessed for their anaemia status in 2014 and 2016, respectively. Children in the earthquake-affected areas had a mean age of 29.5 months and about 40% were less than 24 months old in the 2016 survey (**Table 1**). Just under 10% of women were less than 20 years old and 21.7% did not have formal education. About two-thirds of household heads were male, 23.1% were engaged in business or self-employment, 20% were engaged in agriculture/livestock rearing, and just over half of households owned livestock. Few before-after differences in demographic or socioeconomic characteristics were apparent. Households assessed in both 2014 and 2016 (longitudinal sample) tended to have more educated mothers, were more likely to own livestock, and to have a household head engaged in business or trading compared with those with only pre-earthquake data (**S1 Table**).

**Table 1:** Demographic and socioeconomic characteristics of households, women and children in the baseline and follow-up (cross-sectional).

	Before earthquake (2014)	After earthquake (2016)	p value
**Total number of households (hh)**	982	1056	
**Total number of women**	1015	1083	
**Total number of children under 5 years**	883	998	
**Head of household (HoH), (%)**			0.991
**Male**	64.5	64.5	
**Female**	35.5	35.5	
**Average age of HoH, mean (SD)**	38.9 (15.5)	38.8 (14.8)	0.728
**Occupation of HoH, (% hh)**			0.528
**Agriculture/ livestock/ poultry/ aquaculture**	22.1	20.0	
**Business/ trader /self-employment**	23.0	23.1	
**Wage employment/ salaried Worker**	29.1	29.6	
**Non-earning occupation (housewife/ FCHV)**	18.4	19.7	
**Not working/ retired**	6.2	6.8	
**Student/ other**	1.2	0.8	
Area of land owned by households (hectares), (% hh) ^***^			<0.001
**> 0.5 ha**	16.3	16.1	
**≤ 0.5 ha**	41.7	50.7	
**None**	42.1	33.2	
**Livestock ownership, (% of households)**	54.2	51.1	0.103
Household produced rice (% hh) ^**^	24.1	17.6	0.001
**Households below 20% of the lowest wealth quintile†, (% hh)**	6.5	7.2	0.694
**Household received remittances in past year, (% hh)**	44.2	42.4	0.524
**Remittance received in USD, median (IQR) ‡**	1200 (2000)	1500 (2555)	0.158
**Average age of women, mean (SD)**	26.6 (6.6)	27.2 (6.5)	0.168
Average maternal education, mean of schooling (SD) ^***^	6.6 (4.8)	7.6 (4.9)	<0.001
Women's Dietary Diversity (MDD-W^§^ ≥5), (% of women) ^***^	42.8	50.6	<0.001
Total children, (% of children) ^***^			0.459
**<6 months**	8.8	7.6	
**6–11 months**	10.7	10.7	
**12–23 months**	21.6	21.2	
**24–59 months**	58.9	60.4	
**Predominant breastfeeding (% children <6 months)**	39.7	48.7	0.248
**Prelacteal fed (% children <12 months)**	30.8	30.9	0.982
Breastfed within 1 hour of birth (% children <12 months) ^**^	37.2	52.5	0.001
**Colostrum fed (% children <12 months)**	94.8	93.4	0.736

† Calculated using national data

‡ Exchange rates: 1US Dollars = 100 Nepalese Rupees

§ Minimum Dietary Diversity for Women (MDD-W) calculated using a 24-hour recall period

* p-value <0.05

** p-value <0.01

*** p-value <0.001 for differences between 2014 and 2016

**Fig 2:**
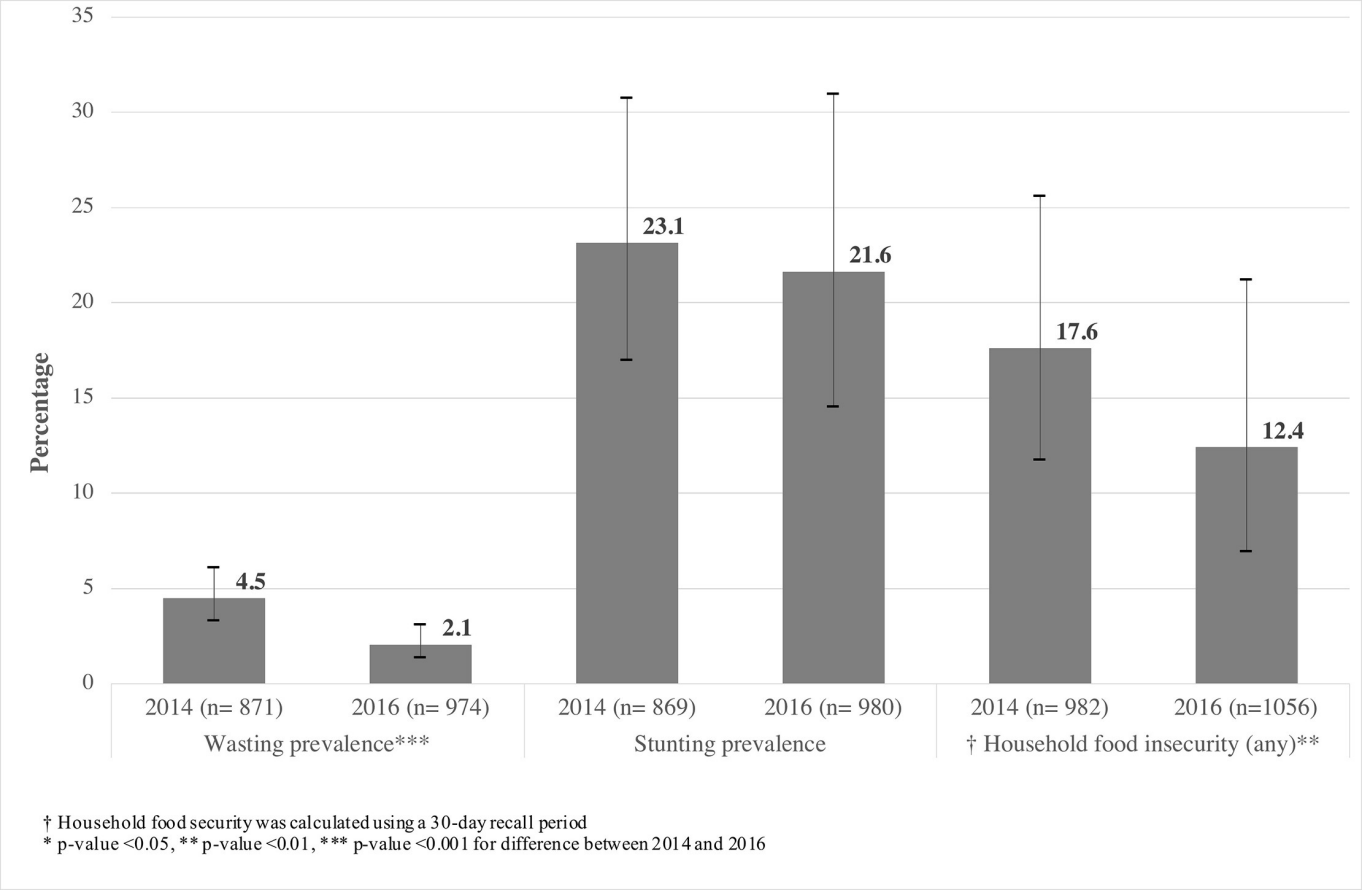
Prevalence of any household food insecurity before, and wasting and stunting in children under five years and (2014) and after (2016) the earthquake in affected areas.

The prevalence of wasting declined significantly from 2014 to 2016 (**Fig 2**) but mean weight-for-height z-scores did not change from -0.4 (-0.5 –-0.3) to -0.3 (-0.4 –-0.1). Severe wasting (<-3 z-scores) were also similar from 0.5% (0.1–1.6) to 0.2% (0.0–1.1). The prevalence of under-five stunting did not change over time, nor did mean height-for-age z-scores (HAZ): -1.1 (95% CI -1.3 –-0.8) vs. -1.0 (-1.2 –-0.7). There was no suggestion of any deterioration in any age subgroup from 2014 vs. 2016 (**S2 Table**). The prevalence of anaemia in children under five years increased from 2014 to 2016 from 37.7% (95% CI 28.4–48.0) to 45.5% (36.4–55.0) but this change was not statistically significant. Similarly, there was no difference in women’s anaemia rates over time: 27.7% (95% CI 16.5–42.6) in 2014 vs. 27.9% (20.0–37.5) in 2016.

The characteristics of households included in the pre-earthquake sample who were accounted for post-earthquake are compared with characteristics of those who were censored due to having moved away during the peri-quake period **(S3 Table)**. Households who moved were more engaged in wage and salaried employment, owned less land, and from higher wealth quintiles, reflecting more urban characteristics. A disproportionately high proportion of HHs that moved out of the sample (73%) came from Gokarneshwor, a peri-urban VDC located in the Kathmandu Valley (Gokarneshwor), that had contributed 48.9% of all households to the 2014 earthquake-affected sample. Children of households that moved were also better off nutritionally prior to the earthquake compared with those who remained, although only the 0.17 z-score increment in weight-for-height was statistically significant (p<0.01).

A higher prevalence of reported shocks was evident in 2016 compared to 2014 (**Table 2**). Nearly half of all sampled households in 2016 in affected VDCs reported damage to their house compared with <2% during the year prior to the earthquake, and most damage was attributed to the quake. Other shocks with increased occurrence reported following the vs. a year before included crop or animal losses (each experienced by about a fifth of households), and business loss (experienced by nearly 10% of households). Over 6% of households lost a family member in the year following the earthquake, a rate that was about double the year before the quake, and 16% reported injury. Among households with longitudinal data, those in the higher wealth quintiles faced more business failures, while households in lower wealth quintiles reported more structural damages, and animal and crop losses (**S4 Table)**.

**Table 2:** Percentage of households experiencing shocks in previous year by standardized recall module before (2014) and after (2016) the earthquake in affected areas.

Reported shocks	2014 (n = 982)	2016 (n = 1056)	
	Any shocks	Any shocks	Attributed to earthquake^†^
	%	%	%
Structure/house damaged	0.5	45.7	45.5
Household lost any of the crops	0	20.5	7.4
Family member faced loss in business	0.5	9.3	6.9
Family member suffered an accident/injury/from a critical illness ^‡^	8.5	-	-
Family member injured	-	16.0	5.9
Family member suffered from any illness	-	8.3	0.7
Household lost any of the livestock/poultry	0.9	21.1	5.6
Family member died	2.5	6.4	2.4
Family member lost job	0.2	3.2	2.2
Household faced legal problem	0.4	1.1	0.1

‡ Disaggregated into injury and illness for 2016

† Reported as absolute proportion of households that reported each category of shock

**Fig 3:**
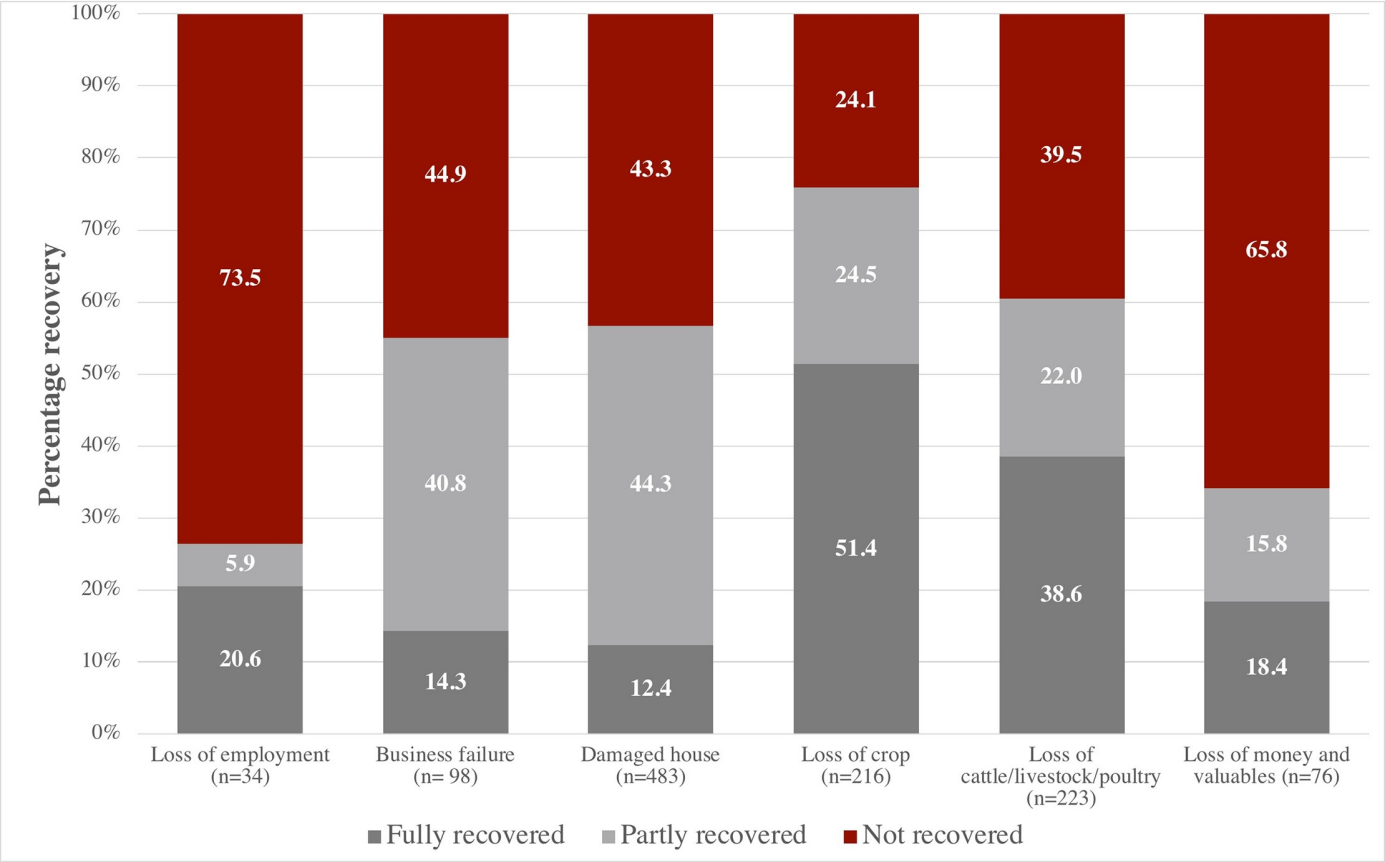
Post-earthquake recovery of households in earthquake-affected VDCs (N = 1056 households).

A year after the earthquake, less than half of all households reported having fully recovered from most shocks, with the exception of crop loss **(Fig 3**). More than 60% of households described having had at least partial recovery from loss of livestock (Fig 3) and there were no significant differences in household ownership of cattle, poultry and goats before and after the quake (**S5 Table**). Nearly 90% of those who experienced housing or structural damage reported partial or non-recovery (Fig 3). Among households in the longitudinal sample, 61% of households in the highest wealth quintile reported not having recovered from structural damage vs. much lower proportions among lower quintiles (range 12.0–27.8%), p<0.001 (**S6 Table**). Similar proportions (42–44%) of households reported receiving remittances before vs. earthquake, though the amount received was slightly higher in 2016, the increase was not significant (p = 0.16) (**Table 1).**

Household food insecurity significantly decreased (p<0.01) from 17.6% (95% CI 11.7–25.6) in 2014 to 12.4% (6.9–21.2) in 2016 (**Fig 2**). Dietary quality also improved, with the proportion of women with minimum dietary diversity score of ≥ 5, rising from 42.8% to 50.6% (p<0.001) after the earthquake (**Table 1**). The proportion of predominantly breastfed infants exhibited an increasing trend, from 39.7% (95% CI 19.8–63.8) in 2014 to 48.7% (32.5–65.2) in 2016, although the difference was not statistically significant. A rise was also seen in the proportion of infants who were breastfed within an hour of birth (p<0.01) from 37.2% (95% CI 25.1–51.1%) of children in 2014 to 52.5% (39.4–65.2%) in 2016 (**Table 1**). We also observed significant improvements in certain handwashing behaviours and water treatment (**S7 Table**). An examination of the proportion of households producing major staple crops and the amounts produced in prior years suggested that proportion of households growing each type of crop decreased in 2016 compared with prior years, but the absolute amount produced by those who grew was similar (**S2 Fig**).

## Discussion

Several analyses of the situation following the 2015 earthquake have been published in the grey literature [7, 22, 23], but to our knowledge this is the first published quantitative comparison of the nutrition and food security situation in the hardest hit areas before vs. after the quake. Consistent with many reports, our findings suggest that households had sustained severe damages, and that few had fully recovered a year after the quake. Despite these shocks, we found that most indicators related to nutrition were stable or even improved compared with their pre-earthquake situation. Specifically, child wasting, the main indicator used to evaluate nutritional status in emergencies, declined from pre-earthquake levels. Child stunting was also slightly lower than before the earthquake. However, the prevalence of both of these indicators prior to the earthquake was much lower in the affected areas than the rest of the country [13], which may be anticipated given that the sample included a dense, peri-urban area in the Kathmandu Valley.

What might explain the fact that nutritional status did not deteriorate as a result of the earthquake, even in the most-affected locations? One possibility is that relief and rehabilitation efforts as well as ongoing programs may have been effective in at least stabilizing the nutritional situation. The Nutrition Cluster response included a number of interventions including promotion of IYCF practices, preventing distribution of breastmilk substitutes, distributing micronutrient powder, and screening and referral of cases of severe acute malnutrition delivered through a “Child Nutrition Week” approach [6]. Reportedly, high coverage was attained of all these services in the immediate aftermath of the quake [6]. Five of the seven affected areas in our sample also had major district-wide, multi-pronged nutrition programs with intensive behaviour change communication interventions related to infant and young child feeding that were ongoing prior to the earthquake and that continued in its aftermath [24]. The improvements we observed in a number of breastfeeding and handwashing behaviours over the two years suggest that these approaches might have been effective in helping mitigate the effects of the earthquake on nutritional status, although our study design limits our ability to attribute changes to those programs or pathways.

Households may have also found other ways to mitigate the impact of shocks on their food security and nutrition. Immediately following the earthquake, there were serious concerns that farmers might not be able to plant their crops in time, resulting in a food security crisis [7]. Such a crisis appeared to have been averted, as our analysis suggests that agricultural productivity of major crops after the earthquake was similar to before, and there was little change in livestock ownership. Threats to the food supply on a population level appeared to be transient and resolved by the time of our follow-up survey–a conclusion that is consistent with other post-earthquake reports suggesting recovery in the agriculture sector [4, 25]. Households also appeared able to maintain similar dietary quality following the earthquake as they had before, supported by findings on dietary diversity and anaemia. This is an important finding given that a common coping strategy during crisis is to reduce the consumption of more expensive foods, particularly animal source foods [26, 27]. We lacked data to explore the use of household loans, which reportedly were an important source of funding to rebuild structures [4] but our data showed no change in remittances from before to after the earthquake, which is consistent with other reports [28, 29].

Our analysis suggests that the nutritional improvements were not due to selection bias caused by the migration of worse-off households out of the crisis-affected areas that we assessed. Indeed, our sensitivity analyses suggested the opposite: households who moved were more likely to be better off socio-economically and to have lived in the Kathmandu Valley cluster.

Most studies of earthquake impacts have focused on short-term consequences like mortality [30] or injury [31, 32]. However, there is a small but growing body of evidence studying the effects of earthquakes on nutrition, derived largely from South American contexts. Studies following the earthquakes in Colombia (1999) and Peru (2001) documented adverse age-cohort effects on linear growth among children born immediately after earthquakes [14, 15]. Although we were not able to find similar age-cohort patterns in our data, it is possible that more time is needed for such patterns to become apparent. Our study provides one of the first examinations of the impact of earthquakes on nutrition in a low income Asian context, made possible by the presence of a system that allowed for more frequent data collection, comparable cross-sectional samples with a longitudinal component, and a wide range of indicators to facilitate detailed examination of nutrition and its influencing factors [33].

One limitation is that our findings, which reflect a *post hoc* sample, have uncertain generalizability to the rest of the government-defined earthquake-affected areas, particularly given that the majority of households in our sample came from within the Kathmandu Valley—an area that is better off nutritionally and economically than most the country. Our inclusion criteria, which focused the survey on households with young children or recently married women, also mean that our household level findings may not be generalizable to all households living in these areas. Recall bias is also a possibility, as data was collected a year after the earthquake, though the direction of such bias is undetermined. It is also possible that nutritional status, particularly wasting or anaemia which are both responsive to shocks in the short term, could have risen immediately after the earthquake but fallen by the time we conducted our survey.

### Conclusions and next steps

Despite the earthquake having devastating effects on many aspects of life in Nepal, we found that many indicators of nutrition and food security were similar or improved a year after compared with the year before the earthquake. Future studies are needed to understand potential longer-term impacts associated with erosion of assets or livelihoods, and cohort effects on the nutrition of children.

## Supporting information

S1 Fig Data flow diagram for nationally representative, mid-year surveys, including the subset of earthquake-affected sampled VDCs, conducted in 2014 and 2016. (DOCX)

S2 Fig Average production of staple crops as reported by households during the year before the mid-2014 and 2016 surveys in earthquake-affected areas. (DOCX)

S1 Table Baseline characteristics of households assessed in 2014 by their post-earthquake follow-up status in 2016. (DOCX)

S2 Table Stunting and mean height-for-age z-scores among children under five years at the times of surveys conducted before (2014) and after (2016) the earthquake in affected areas. (DOCX)

S3 Table Baseline characteristics of households assessed in 2014 by their post-earthquake censoring due to having moved from their recorded residence or lost to follow-up by 2016. (DOCX)

S4 Table Shocks reported by households in affected areas (longitudinal sample) in 2016 during the year following the earthquake according to their pre-earthquake socioeconomic (SES) status defined by a wealth index classification in 2014. (DOCX)

S5 Table Types of livestock ownership by households at the times of mid-year surveys conducted before (2014) and after (2016) the earthquake in affected areas. (DOCX)

S6 Table Recovery in households that experienced shocks according to their pre-earthquake 2014 socioeconomic (SES) status. (DOCX)

S7 Table Knowledge of when hands should be washed with soap and water in households with children under 5 years at the times of mid-year surveys before (2014) and after (2016) the earthquake in affected areas. (DOCX)
