# Supplementary material for: Nutritional resilience in Nepal following the earthquake of 2015
Source: PLoS One. 2018 Nov 7;13(11):e0205438. doi: 10.1371/journal.pone.0205438 (PMC6221269; doi:10.1371/journal.pone.0205438)

**S1 Figure. Data flow diagram for nationally representative, mid-year surveys, including the subset of earthquake-affected sampled VDCs, conducted in 2014 and 2016**


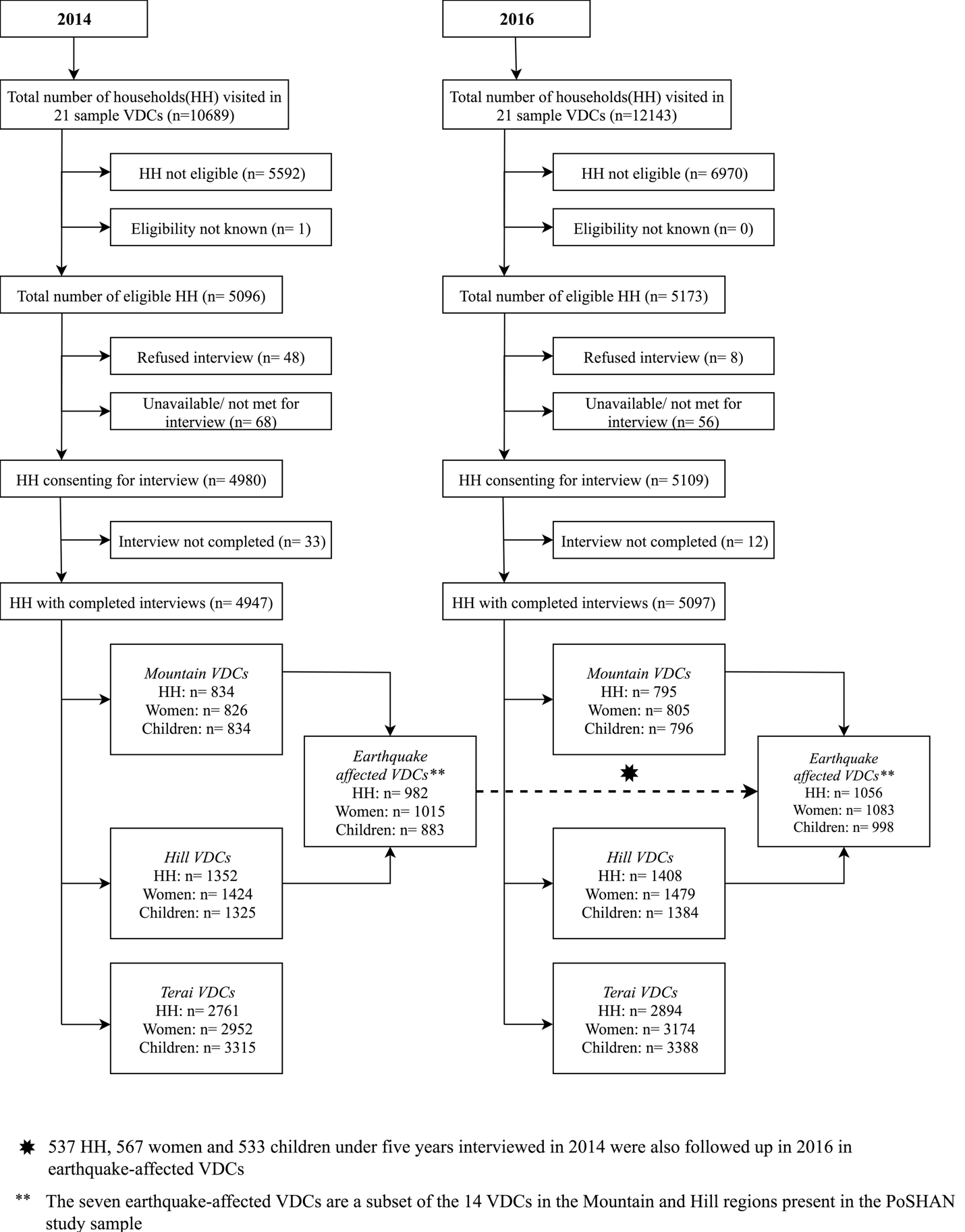

Supplement: S1 Fig — (DOCX) [file pone.0205438.s001.docx]
