# Supplementary material for: Nutritional resilience in Nepal following the earthquake of 2015
Source: PLoS One. 2018 Nov 7;13(11):e0205438. doi: 10.1371/journal.pone.0205438 (PMC6221269; doi:10.1371/journal.pone.0205438)
Supplement: S2 Fig — (DOCX) [file pone.0205438.s002.docx]

**S2 Figure. Average production of staple crops as reported by households during the year before the mid-2014 and 2016 surveys in earthquake-affected areas**
