# Supplementary material for: Nutritional resilience in Nepal following the earthquake of 2015
Source: PLoS One. 2018 Nov 7;13(11):e0205438. doi: 10.1371/journal.pone.0205438 (PMC6221269; doi:10.1371/journal.pone.0205438)
Supplement: S1 Table — (DOCX) [file pone.0205438.s003.docx]

**S1 Table. Baseline characteristics of households assessed in 2014 by their post-earthquake follow-up status in 2016**

|  | Assessed only in 2014 | Assessed in 2014 & 2016 | p value |
| --- | --- | --- | --- |
| Total number of households (hh) | 445 | 537 |  |
| Total number of women | 448 | 567 |  |
| Total number of children under 5 years | 350 | 533 |  |
| Head of household (HoH), (%) |  |  | 0.876 |
| Male | 64.0 (50.3 to 75.8) | 64.8 (28.4 to 42.6) |  |
| Female | 36.0 (24.2 to 49.7) | 35.2 (57.4 to 71.6) |  |
| Average age of HoH ^**^ | 36.5 (32.6 to 40.5) | 40.9 (37.6 to 44.2) | 0.001 |
| Occupation of HoH, (% hh) ^***^ |  |  | <0.001 |
| Agriculture/ livestock/ poultry/ aquaculture | 16.4 (6.4 to 36.1) | 26.8 (11.6 to 50.7) |  |
| Business/ trader /self-employment | 26.5 (17.7 to 37.7) | 20.1 (11.7 to 32.4) |  |
| Wage employment/ salaried Worker | 33.0 (25.3 to 41.8) | 25.7 (17.3 to 36.4) |  |
| Non-earning occupation (housewife/ FCHV) | 18.4 (111.9 to 27.4) | 18.4 (13.9 to 24.0) |  |
| Not working/ retired | 4.3 (2.1 to 8.3) | 7.8 (4.5 to 13.3) |  |
| Student/ other | 1.3 (0.7 to 2.5) | 1.1 (0.4 to 3.4) |  |
| Area of land owned by households (hectares), (% hh) |  |  | 0.170 |
| > 0.5 ha | 64.5 (57.3 to 71.1) | 58.3 (50.7 to 65.5) |  |
| ≤ 0.5 ha | 18.2 (9.9 to 31.2) | 20.9 (9.0 to 41.2) |  |
| None | 17.3 (10.8 to 26.5) | 20.9 (11.8 to 34.2) |  |
| Livestock ownership, (% of households) ^***^ | 44.9 (20.3 to 72.3) | 61.8 (28.9 to 86.6) | <0.001 |
| Household received remittances in past year, (% hh) | 42.9 (32.2 to 54.3) | 45.3 (31.8 to 59.5) | 0.593 |
| Remittance received in USD, median (IQR) ‡ | 1420 (2500) | 1050 (1900) | 0.896 |
| Household wealth quintiles†, (% hh) ^**^ |  |  | 0.001 |
| Lowest | 5.8 (2.1 to 15.8) | 7.1 (2.6, 18.0) |  |
| Low | 6.1 (2.2 to 15.9) | 7.3 (2.6, 18.6) |  |
| Middle | 16.9 (6.0 to 39.1) | 24.6 (9.1, 51.6) |  |
| High | 13.0 (6.2 to 25.2) | 15.5 (7.8, 28.3) |  |
| Highest | 58.2 (26.5 to 84.3) | 45.6 (14.8, 80.2) |  |
| Any household food insecurity, (% hh) | 18.2 (13.1 to 24.6} | 17.1 (10.4, 27.0) | 0.581 |
| Average age of women | 26.8 (25.7 to 28.0) | 26.4 (25.4, 27.4) | 0.528 |
| Average maternal education (years of schooling) ^*^ | 6.0 (5.1 to 6.9) | 7.1 (5.5, 8.8) | 0.017 |
| Women's Dietary Diversity (MDD-W^§^ ≥5), (% of women) | 44.0 (31.7 to 57.0) | 41.8 (26.7, 58.6) | 0.459 |
| Total children, (% of children) ^**^ |  |  | 0.003 |
| <6 months | 8.9 (6.2, 12.5) | 8.8 (6.5, 11.8) |  |
| 6-11 months | 9.7 (7.2, 12.9) | 11.3 (9.3, 13.6) |  |
| 12-23 months | 18.0 (15.2, 21.2) | 24.0 (20.6, 27.8) |  |
| 24-59 months | 63.4 (58.8, 67.8) | 55.9 (51.6, 60.1) |  |
| Predominant breastfeeding (% children <6 months) | 35.5 (16.3, 60.9) | 42.6 (19.4, 69.5) | 0.412 |
| Prelacteal fed (% children <12 months) | 27.7 (19.4, 37.9) | 32.7 (21.1, 46.9) | 0.305 |
| Breastfed within 1 hour of birth (% children <12 months) | 35.4 (19.8, 54.8) | 38.3 (27.1, 50.9) | 0.593 |
| Colostrum fed (% children <12 months) | 93.8 (85.0, 97.6) | 95.3 (82.9, 98.8) | 0.599 |
| Prevalence of wasting, (%children <60 months) | 2.9 (1.0, 8.0) | 5.5 (4.4, 6.9) | 0.202 |
| Mean WHZ ^**^ | -0.3 (-0.4, -0.1) | -0.5 (-0.6, -0.4) | 0.003 |
| Prevalence of stunting, (%children <60 months) | 20.5 (16.7, 24.9) | 24.9 (16.1, 36.3) | 0.331 |
| Mean HAZ | -1.0 (-1.2, -0.9) | -1.1 (-1.4, -0.8) | 0.656 |

† Calculated using national data

‡ Exchange rates: 1US Dollars =100 Nepalese Rupees

§ Minimum Dietary Diversity for Women (MDD-W) calculated using a 24-hour recall period

* p-value <0.05, ** p-value <0.01, *** p-value <0.001 for differences between HH assessed only in 2014 and those assessed in both 2014 and 2016
