# Supplementary material for: Nutritional resilience in Nepal following the earthquake of 2015
Source: PLoS One. 2018 Nov 7;13(11):e0205438. doi: 10.1371/journal.pone.0205438 (PMC6221269; doi:10.1371/journal.pone.0205438)
Supplement: S2 Table — (DOCX) [file pone.0205438.s004.docx]

**S2 Table. Stunting and mean height-for-age z*-*scores among children under five years at the times of surveys conducted before (2014) and after (2016) the earthquake in affected areas**

|  |  |  |  |  |  |  |  |  |  |
| --- | --- | --- | --- | --- | --- | --- | --- | --- | --- |
| Age categories | **Total children** | **Mean HAZ (95%CI)** | **Prevalence of stunting† (% (95%CI)** |  | **Total children** | **Mean HAZ (95%CI)** | **Prevalence of stunting† (%) (95%CI)** | **p value for mean HAZ** | **p value for prevalence of stunting** |
| <6 months | 78 | 0.3 (-0.0 to 0.6) | 2.6 (0.4 to 14.1) |  | 76 | 0.8 (0.6 to 0.9) **^*^** | 1.4 (0.3 to 5.3) | 0.033 | 0.621 |
| 6-11 months | 94 | -0.4 (-0.9 to 0.2) | 8.6 (4.1 to 17.0) |  | 107 | -0.2 (-0.6 to 0.2) | 6.5 (3.7 to 11.3) | 0.153 | 0.562 |
| 12-17 months | 97 | -0.7 (-1.1 to -0.4) | 17.7 (11.8 to 25.8) |  | 117 | -0.6 (-1.0 to -0.2) | 8.4 (4.4 to 15.6) **^*^** | 0.306 | 0.021 |
| 18-23 months | 94 | -1.0 (-1.3 to -0.8) | 17.4 (10.1 to 28.3) |  | 94 | -1.0 (-1.4 to -0.7) | 22.6 (13.6 to 35.2) | 0.777 | 0.364 |
| 24-59 months | 520 | -1.5 (-1.7 to -1.3) | 30.9 (23.2 to 39.8) |  | 603 | -1.3 (-1.7 to -1.0) | 28.8 (18.8 to 41.6) | 0.301 | 0.559 |
| Total | 883 | -1.1 (-1.3 to -0.8) | 23.1 (17.0 to 30.7) |  | 998 | -1.0 (-1.2 to -0.7) | 21.6 (14.5 to 30.9) | 0.064 | 0.466 |

† Proportion of children that fall under -2 HAZ

p-value *<0.05, ** <0.01, ***<0.001 for differences in mean HAZ and proportion between 2014 and 2016 within each age category
