# Supplementary material for: Nutritional resilience in Nepal following the earthquake of 2015
Source: PLoS One. 2018 Nov 7;13(11):e0205438. doi: 10.1371/journal.pone.0205438 (PMC6221269; doi:10.1371/journal.pone.0205438)
Supplement: S3 Table — (DOCX) [file pone.0205438.s005.docx]

**S3 Table. Baseline characteristics of households assessed in 2014 by their post-earthquake censoring due to having moved from their recorded residence or lost to follow-up by 2016**

| 2014 Characteristics | Accounted for in 2016^Ω^ | Censored due to having moved in 2016 ^Ω^ | p value |
| --- | --- | --- | --- |
| Total number of households (hh) | 741 | 241 |  |
| Total number of women | 772 | 243 |  |
| Total number of children under 5 years | 671 | 212 |  |
| Head of household (HoH), (% hh) |  |  | 0.404 |
| Male | 63.3 (56.2, 69.9) | 68.0 (50.9, 81.4) |  |
| Female | 36.7 (30.1, 43.8) | 32.0 (18.6, 49.1) |  |
| Average age of HoH ^***^ | 41.5 (38.4, 44.5) | 31.2 (29.8, 32.6) | <0.001 |
| Occupation of HoH, (% hh) ^***^ |  |  | <0.001 |
| Agriculture/ livestock/ poultry/ aquaculture | 27.5 (13.1, 48.9) | 5.4 (1.4, 18.3) |  |
| Business/ trader /self-employment | 20.8 (12.8, 31.9) | 29.9 (20.8, 40.9) |  |
| Wage employment/ salaried Worker | 25.1 (17.3, 34.9) | 41.1 (36.9, 45.4) |  |
| Non-earning occupation (housewife/ FCHV) | 18.5 (14.1, 23.8) | 18.3 (10.0, 31.0) |  |
| Not working/ retired | 7.0 (4.1, 11.7) | 3.7 (1.2, 10.9) |  |
| Student/ other | 1.1 (0.4, 3.3) | 1.7 (0.9, 3.2) |  |
| Area of land owned by households (hectares), (% hh) ^***^ |  |  | <0.001 |
| > 0.5 ha | 19.6 (10.9, 32.6) | 6.2 (4.1, 9.4) |  |
| ≤ 0.5 ha | 45.1 (37.7, 52.7) | 31.1 (26.6, 36.0) |  |
| None | 35.4 (29.9, 41.3) | 62.7 (57.1, 67.9) |  |
| Livestock ownership, (% of households) ^***^ | 63.7 (32.9, 86.3) | 24.9 (10.0, 49.6) | <0.001 |
| Household received remittances in past year, (% hh) | 45.8 (32.9, 59.3) | 39.4 (30.9, 48.6) | 0.220 |
| Remittance received in USD, median (IQR) ‡ | 1000 (2000) | 1500 (2420) | 0.110 |
| Household wealth quintiles†, (% hh) ^***^ |  |  | <0.001 |
| Lowest | 7.8 (3.1, 18.6) | 2.5 (0.5, 10.7) |  |
| Low | 8.1 (3.4, 18.3) | 2.5 (0.5, 11.7) |  |
| Middle | 25.0 (10.2, 49.5) | 9.1 (2.5, 27.9) |  |
| High | 15.9 (8.7, 27.4) | 9.5 (3.4, 23.9) |  |
| Highest | 43.2 (14.8, 76.8) | 76.3 (46.4, 92.3) |  |
| Any household food insecurity, (% hh) | 17.5 (11.2, 26.4) | 17.8 (12.2, 25.3) | 0.919 |
| Average age of women ^***^ | 27.0 (26.2, 27.9) | 25.1 (24.5, 25.8) | <0.001 |
| Average maternal education (years of schooling) | 6.7 (5.2, 8.2) | 6.4 (5.8, 7.0) | 0.575 |
| Women's Dietary Diversity (MDD-W^§^ ≥5), (% of women) ^*^ | 41.1 (27.2, 56.6) | 48.1 (36.6, 59.0) | 0.039 |
| Total children, (% of children) ^***^ |  |  | <0.001 |
| <6 months | 8.2 (6.2, 10.8) | 10.8 (8.2, 14.2) |  |
| 6-11 months | 9.5 (7.9, 11.5) | 14.2 (11.9, 16.7) |  |
| 12-23 months | 21.5 (18.8, 24.4) | 22.2 (18.3, 26.6) |  |
| 24-59 months | 60.8 (56.6, 64.8) | 52.8 (50.1, 55.5) |  |
| Predominant breastfeeding (% children <6 months) | 40.0 (20.0, 64.0) | 39.1 (12.7, 74.0) | 0.917 |
| Prelacteal fed (% children <12 months) | 33.6 (21.5, 48.3) | 24.5 (17.1, 33.9) | 0.070 |
| Breastfed within 1 hour of birth (% children <12 months) | 38.7 (27.8, 50.8) | 34.0 (17.4, 55.7) | 0.430 |
| Colostrum fed (% children <12 months) | 95.0 (80.8, 98.8) | 94.3 (85.2, 98.0) | 0.880 |
| Prevalence of wasting, (%children <60 months) | 5.3 (4.0, 6.9) | 1.9 (0.4, 8.2) | 0.139 |
| Mean WHZ ^*^ | -0.4 (-0.6, -0.3) | -0.3 (-0.4, -0.1) | 0.038 |
| Prevalence of stunting, (%children <60 months) | 24.4 (17.0, 33.6) | 19.3 (13.2, 27.4) | 0.325 |
| Mean HAZ | -1.1 (-1.4, -0.8) | -0.94 (-1.2, -0.7) | 0.287 |

^Ω^ Accounted for category includes HH who were included in the 2016 survey, merged or split households, and ineligible households with all children >71 months. HH that moved permanently or temporarily out of the study wards/PoSHAN areas, or who were not found in the 2016 survey are censored due to their move status.

† Calculated using national data

‡ Exchange rates: 1US Dollars =100 Nepalese Rupees

§ Minimum Dietary Diversity for Women (MDD-W) calculated using a 24-hour recall period

* p-value <0.05, ** p-value <0.01, *** p-value <0.001 for differences between households that were accounted for in 2016 and those that were censored
