# Supplementary material for: Nutritional resilience in Nepal following the earthquake of 2015
Source: PLoS One. 2018 Nov 7;13(11):e0205438. doi: 10.1371/journal.pone.0205438 (PMC6221269; doi:10.1371/journal.pone.0205438)
Supplement: S4 Table — (DOCX) [file pone.0205438.s006.docx]

**S4 Table. Shocks reported by households in affected areas (longitudinal sample) in 2016 during the year following the earthquake according to their pre-earthquake socioeconomic (SES) status defined by a wealth index classification in 2014**

| 2014 SES  quintile^‡^ | Number of households | | Shocks reported due to earthquake (% of households in each SES categories) | | | | | | | |
| --- | --- | --- | --- | --- | --- | --- | --- | --- | --- | --- |
|  |  |  | **Death** | **Injury** | **Structural damage^***^** | **Crop loss^***^** | **Animal loss^***^** | **Job**  **loss** | **Business failure^***^** | **Cash**  **loss** |
| Lowest | | 38 | 0.0 | 10.5 | 65.8 | 18.4 | 13.2 | 2.6 | 0.0 | 2.6 |
| Low | | 39 | 2.6 | 5.1 | 64.1 | 20.5 | 15.4 | 0.0 | 2.6 | 7.7 |
| Middle | | 132 | 0.0 | 5.3 | 47.7 | 11.4 | 9.1 | 3.0 | 3.8 | 0.0 |
| High | | 83 | 0.0 | 4.8 | 43.4 | 4.8 | 4.8 | 2.4 | 4.8 | 2.4 |
| Highest | | 245 | 2.5 | 3.7 | 40.8 | 2.9 | 0.8 | 0.8 | 10.6 | 1.6 |
| Total | | 537 | 1.3 | 4.8 | 46.4 | 7.6 | 5.4 | 1.7 | 6.7 | 1.9 |
| p value | |  | 0.131 | 0.107 | <0.001 | <0.001 | <0.001 | 0.330 | <0.001 | 0.374 |

p-value for trend *<0.05, ** <0.01, ***<0.001 for each type of shock experienced by households

^‡^ Analysis was conducted among households that were in the longitudinal sample (n=537), i.e., that were assessed in both 2014 & 2016
