# Supplementary material for: Nutritional resilience in Nepal following the earthquake of 2015
Source: PLoS One. 2018 Nov 7;13(11):e0205438. doi: 10.1371/journal.pone.0205438 (PMC6221269; doi:10.1371/journal.pone.0205438)
Supplement: S5 Table — (DOCX) [file pone.0205438.s007.docx]

**S5 Table. Types of livestock ownership by households at the times of mid-year surveys conducted before (2014) and after (2016) the earthquake in affected areas**

|  | **2014** | **2016** | **p value** |
| --- | --- | --- | --- |
| Total HH | 982 | 1,056 |  |
| HH that own livestock^†^, (%) | 54.2 | 51.1 | 0.103 |
| HH own cattle^‡^ | 57.5 | 54.3 | 0.688 |
| Own 1-3 cattle (%HH that own cattle) | 74.8 | 73.7 |  |
| Own 4-7 cattle | 21.6 | 22.5 |  |
| Own >8 cattle | 3.6 | 3.8 |  |
| HH own goat^‡^ | 53.5 | 53.4 | 0.094 |
| Own 1-3 goat (%HH that own goat) | 52.8 | 45.1 |  |
| Own 4-7 goat | 39.4 | 38.2 |  |
| Own >8 goat | 7.8 | 16.7 |  |
| HH own poultry^‡^ | 73.8 | 66.2 | 0.889 |
| Own 1-3 poultry (%HH that own poultry) | 32.0 | 30.0 |  |
| Own 4-7 poultry | 29.2 | 31.7 |  |
| Own >8 poultry | 38.9 | 38.4 |  |

† Proportion calculated in all households, ‡ calculated among households that own livestock

* p-value <0.05, ** p-value <0.01, ***<0.001 for differences between 2014 and 2016
