# Supplementary material for: Nutritional resilience in Nepal following the earthquake of 2015
Source: PLoS One. 2018 Nov 7;13(11):e0205438. doi: 10.1371/journal.pone.0205438 (PMC6221269; doi:10.1371/journal.pone.0205438)
Supplement: S6 Table — (DOCX) [file pone.0205438.s008.docx]

**S6 Table. Recovery in households that experienced shocks according to their pre-earthquake 2014 socioeconomic (SES) status**

| Damage to house^***^ | Total Households | % Not recovered | % Partly recovered | % Fully recovered |
| --- | --- | --- | --- | --- |
| Lowest | 25 | 24.0 | 56.0 | 20.0 |
| Low | 25 | 12.0 | 72.0 | 16.0 |
| Middle | 63 | 12.7 | 69.8 | 17.5 |
| High | 36 | 27.8 | 58.3 | 13.9 |
| Highest | 100 | 61.0 | 24.0 | 15.0 |

p-value for trend *<0.05, ** <0.01, ***<0.001

Recovery from injury, losses of animals, crops, job, or cash, and business failure were omitted due to sparse data.
