# Supplementary material for: Nutritional resilience in Nepal following the earthquake of 2015
Source: PLoS One. 2018 Nov 7;13(11):e0205438. doi: 10.1371/journal.pone.0205438 (PMC6221269; doi:10.1371/journal.pone.0205438)
Supplement: S7 Table — (DOCX) [file pone.0205438.s009.docx]

**S7 Table. Knowledge of when hands should be washed with soap and water in households with children under 5 years at the times of mid-year surveys before (2014) and after (2016) the earthquake in affected areas**

|  | 2014 | 2016 | p value |
| --- | --- | --- | --- |
| Total HH with children under 5 years | 767 | 856 |  |
| Total no of mothers with children under 5 years | 783 | 870 |  |
| Proportion of HH with children <5 years that treat water ^***^ |  |  |  |
| Yes | 44.7 | 55.4 | <0.001 |
| Sometimes | 5.5 | 1.5 |  |
| No | 49.8 | 43.1 |  |
| Proportion of mothers with an under-5 years old child who reported washing hands with soap and water | | | |
| Before cooking | 21.3 | 26.8 | 0.110 |
| Before eating ^*^ | 65.4 | 72.0 | 0.015 |
| Before feeding the child ^***^ | 21.2 | 30.3 | <0.001 |
| After going to the toilet | 93.0 | 96.1 | 0.068 |
| After cleaning a child who has defecated | 33.1 | 38.6 | 0.124 |
| After handling chicken, cow, duck or other animal feces ^***^ | 40.0 | 82.8 | <0.001 |

p-values * <0.05, **<0.01, ***<0.001, testing 2014 vs. 2016 for each area
